# New Appliances and Things Medical

**Published:** 1906-01-06

**Authors:** 


### NEW APPLIANCES AND THINGS MEDICAL.

[We shall be glad to receive, at our Office, 28 & 29 Southampton Street, Strand, London, W.C., from the manufacturers, specimens of all new preparations and appliances which may be brought out from time to time.]

### PURE AUSTRALIAN BRANDY.

(H. R. Williams and Company, 6 Lime Street, London, E.C.)

The medical value of brandy is beyond dispute and is emphasised by the fact that the British Pharmacopœia recognises it as a preparation of alcohol and gives it the name of "Spiritus vini gallici." It is significant in this connection to note that the Pharmacopœia calls it a spirit, spirit being the generic term for colourless, or almost colourless, alcoholic solutions containing volatile substances. The Pharmacopœia evidently, therefore, regards brandy not simply as ethylic alcohol, but as containing in addition some volatile substances. It defines brandy as a spirituous liquid distilled from wine and matured by age and containing not less than 36.5 per cent. by weight of ethyl hydroxide. The volatile substances which brandy contains other than ethylic alcohol have been the subject of much dispute—especially is this true of the so-called compound ethers. There can be no doubt that old brandy has a different therapeutical effect to raw spirit, but we are not in this connection prepared to go so far as some authors who affirm that old brandy is almost a pure stimulant and hardly an intoxicant or narcotic at all. If we come to examine the part played in the physiological action of brandy by its different chemical constituents, we begin to tread on very uncertain ground, and herein lies the great difficulty in fixing a chemical standard for brandy. It cannot be maintained that, on the one hand, directly a spirit distilled from wine fails to contain a given portion of compound ether, it ceases to be brandy; nor on the other hand, a spirit derived from some other source directly it contains a given quantity of compound ethers becomes brandy. Nor can it be held that the term brandy must only be applied to the product obtained by the distillation of wine in France. We are afraid in the present state of our knowledge that taste and flavour of the finished product and the materials from which it is made can be our only criterion of what is or what is not brandy. We welcome the advent of an Australian brandy, and the spirit before us is in every sense a genuine brandy, that is a product made by the distillation of Australian wine. A disadvantage under which brandy labours, at any rate from the point of the practitioner in poorer districts, is its expense, and we are glad to see that the brandy before us can be purchased for 3s. 6d. per bottle.

